# Episignature Mapping of *TRIP12* Provides Functional Insight into Clark–Baraitser Syndrome

**DOI:** 10.3390/ijms232213664

**Published:** 2022-11-08

**Authors:** Liselot van der Laan, Kathleen Rooney, Mariëlle Alders, Raissa Relator, Haley McConkey, Jennifer Kerkhof, Michael A. Levy, Peter Lauffer, Mio Aerden, Miel Theunis, Eric Legius, Matthew L. Tedder, Lisenka E. L. M. Vissers, Saskia Koene, Claudia Ruivenkamp, Mariette J. V. Hoffer, Dagmar Wieczorek, Nuria C. Bramswig, Theresia Herget, Vanesa López González, Fernando Santos-Simarro, Pernille M. Tørring, Anne-Sophie Denomme-Pichon, Bertrand Isidor, Boris Keren, Sophie Julia, Elise Schaefer, Christine Francannet, Pierre-Yves Maillard, Mala Misra-Isrie, Hilde Van Esch, Marcel M. A. M. Mannens, Bekim Sadikovic, Mieke M. van Haelst, Peter Henneman

**Affiliations:** 1 Department of Human Genetics, Amsterdam Reproduction & Development Research Institute, Amsterdam University Medical Centers, Meibergdreef 9, 1105 AZ Amsterdam, The Netherlands; 2 Department of Pathology and Laboratory Medicine, Western University, London, ON N5A 3K7, Canada; 3 Verspeeten Clinical Genome Centre, London Health Science Centre, London, ON N6A 5W9, Canada; 4 Department of Pediatric Endocrinology, Emma Children’s Hospital, Amsterdam Gastroenterology, Endocrinology & Metabolism, Amsterdam UMC, University of Amsterdam, 1105 AZ Amsterdam, The Netherlands; 5 Centre for Human Genetics, University Hospitals Leuven, KU Leuven, 3000 Leuven, Belgium; 6 Greenwood Genetic Center, Greenwood, SC 29646, USA; 7 Department of Human Genetics, Radboud University Medical Center, 6525 GA Nijmegen, The Netherlands; 8 Department of Clinical Genetics, Leiden University Medical Center, 2333 ZA Leiden, The Netherlands; 9 Institute of Human Genetics, Medical Faculty, Heinrich-Heine-University, 40225 Düsseldorf, Germany; 10 Institute of Human Genetics, University Medical Center Hamburg-Eppendorf, 20251 Hamburg, Germany; 11 Sección Genética Médica, Servicio de Pediatría, Hospital Clínico Universitario Virgen de la Arrixaca, IMIB-Arrixaca, CIBERER, 30120 Murcia, Spain; 12 Institute of Medical and Molecular Genetics (INGEMM), Hospital Universitario La Paz, IdiPAZ, CIBERER, ISCIII, 28029 Madrid, Spain; 13 Department of Clinical Genetics, Odense University Hospital, 5000 Odense, Denmark; 14 UF6254 Innovation en Diagnostic Genomique des Maladies Rares, 21070 Dijon, France; 15 Équipe Génétique des Anomalies du Développement (GAD), CHU Dijon-Bourgogne, 21000 Dijon, France; 16 Service de Génétique Médicale, CHU de Nantes, 44000 Nantes, France; 17 Department of Medical Genetics, Pitié-Salpêtrière Hospital, AP-HP, Sorbonne Université, 75013 Paris, France; 18 Service de Génétique Clinique, CHU Toulouse, 31300 Toulouse, France; 19 Service de Genetique Medicale, CHU de Clermont-Ferrand, 63000 Clermont-Ferrand, France; 20 Institut Jérôme Lejeune, 75015 Paris, France

**Keywords:** TRIP12, Clark–Baraitser syndrome, intellectual disability, DNA methylation, episignature

## Abstract

Clark–Baraitser syndrome is a rare autosomal dominant intellectual disability syndrome caused by pathogenic variants in the *TRIP12* (Thyroid Hormone Receptor Interactor 12) gene. *TRIP12* encodes an E3 ligase in the ubiquitin pathway. The ubiquitin pathway includes activating E1, conjugating E2 and ligating E3 enzymes which regulate the breakdown and sorting of proteins. This enzymatic pathway is crucial for physiological processes. A significant proportion of *TRIP12* variants are currently classified as variants of unknown significance (VUS). Episignatures have been shown to represent a powerful diagnostic tool to resolve inconclusive genetic findings for Mendelian disorders and to re-classify VUSs. Here, we show the results of DNA methylation episignature analysis in 32 individuals with pathogenic, likely pathogenic and VUS variants in *TRIP12*. We identified a specific and sensitive DNA methylation (DNAm) episignature associated with pathogenic *TRIP12* variants, establishing its utility as a clinical biomarker for Clark–Baraitser syndrome. In addition, we performed analysis of differentially methylated regions as well as functional correlation of the *TRIP12* genome-wide methylation profile with the profiles of 56 additional neurodevelopmental disorders.

## 1. Introduction

Pathogenic variants in *TRIP12* (Thyroid Hormone Receptor Interactor 12; OMIM #604506), located on chromosome 2q36.3, result in an autosomal dominant neurodevelopmental disorder termed Clark–Baraitser syndrome (OMIM #617752) [1]; additionally, *TRIP12* is one of the primary genes linked to intellectual disability (ID) [2]. This is also supported by a meta-analysis that was performed by Lelieveld et al. [3]. ID is the most common developmental disorder and affects 1–3% people worldwide [4]. Mendelian-inherited ID has been linked to over 1400 genes, including *TRIP12*; however, ID is also known as a complex trait [5].

Clark–Baraitser syndrome is characterized by ID, which may be accompanied by autism spectrum disorder (ASD), speech delay and/or obesity [5,6]. Moreover, Clark–Baraitser syndrome can also involve dysmorphic features, including narrow up-slanting palpebral fissures and a distinct mouth with downturned corners [5,7].

*TRIP12* encodes for an E3 ligase of the ubiquitin pathway, which has been implicated in ASD [2,8]. The ubiquitin pathway includes activating E1, conjugating E2 and ligating E3 enzymes. This process regulates the breakdown and sorting of proteins and is crucial for physiological processes such as cell cycle progression, DNA damage repair, chromatin remodeling and cell differentiation [5]. Thus, pathogenic variants in *TRIP12* result in dysfunction of the ubiquitin pathway which, in turn, leads to a wide range of disorders, including intellectual disability, cancer and neurodegenerative diseases such as Parkinson’s and Alzheimer disease [2]. TRIP12 is involved in chromatin remodeling [9] and has been shown to interact with BAF57 (*SMARCE1*), a subunit of the ATP-dependent SWI/SNF (Switch/Sucrose Non-Fermentable) chromatin remodeling complex [5]. *TRIP12* is responsible for the ubiquitination of *SMARCE1* [10]. Dysfunction of other genes involved in the SWI/SNF chromatin remodeling complex has been associated with aberrant DNA methylation patterns [11]. *TRIP12* includes four characterized protein domains: a catalytic HECT (homologous to the E6-AP carboxyl terminus), WWE (tryptophan–tryptophan–glutamate), ARM (armadillo repeats) and an IDR (intrinsically disordered regions) domain, which define the structure and functionality of the TRIP12 protein [5,12,13,14].

Genes that are linked to the epigenetic machinery can be divided in to four categories: genes coding for (1) readers, (2) writers, (3) erasers and (4) remodelers. The phenotypes that result from aberrations in genes involved in the epigenetic machinery are often linked to neurodevelopmental features such as intellectual disability, growth retardation and limb/nail abnormalities [9,15]. It has been shown that aberrations in genes encoding for proteins that function in epigenetic regulation have a pivotal role in cell differentiation processes and are, in particular, critical during embryonic and fetal development. Consequently, variants in genes of the epigenetic machinery can lead to errors in epigenetic patterning and concomitantly with contextually inappropriate gene expression that, generally, show unique DNA methylation patterns, known as episignatures [16]. Episignatures can be used as sensitive and specific biomarkers for an expanding number of neurodevelopmental disorders [17,18,19,20]. Currently, over 55 episignatures have been described, associated with 65 disorders [21,22]. Episignatures are used for diagnostic clinical testing [23] and for classification of variants of uncertain significance (VUS) [22]. DNA methylation profiling can also provide insights into the molecular etiology in these diseases. The clinical phenotype and TRIP12 protein function overlap that of the previously described DNAm episignature disorders, which led us to hypothesize the existence of a *TRIP12*-related episignature.

In this study, we aim to identify and validate a DNA methylation episignature for *TRIP12*, use it to reclassify genetic VUSs in a patient cohort, and assess the functional genetic features of genome-wide DNA methylation changes in this disorder.

## 2. Results

### 2.1. Identification and Assessment of an Episignature for TRIP12

**Figure 1:**
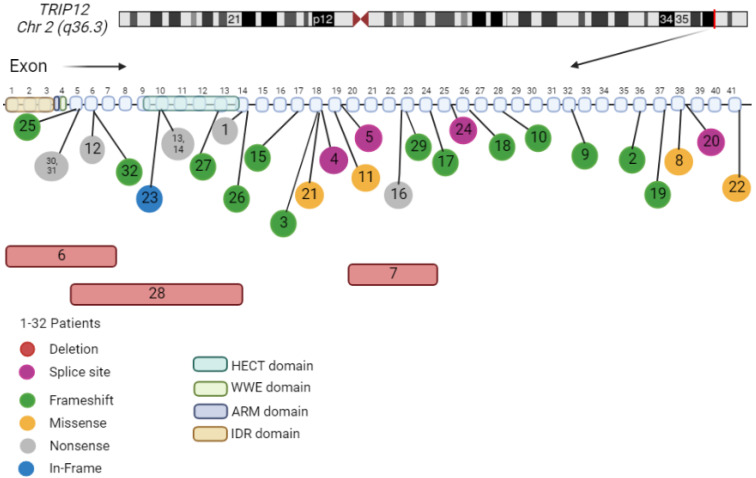
Individuals’ genetic information. For corresponding variant information, see Table 1. Cases with deletions (red square), splice site (purple circle), frameshift (green round), missense (yellow circle) and nonsense (grew circle) variants. Alamut Visual version 1.6.1 NM_004238.3 TRIP12. Created with Biorender.com (accessed on 3 March 2022) [5,24].

The molecular details at diagnosis and demographics of our cohort of 32 patients are summarized (Figure 1 and Table 1). All individuals carried *TRIP12* variants (deletion, splice site, frameshift, missense, nonsense or in-frame variants).

**Figure 2:**
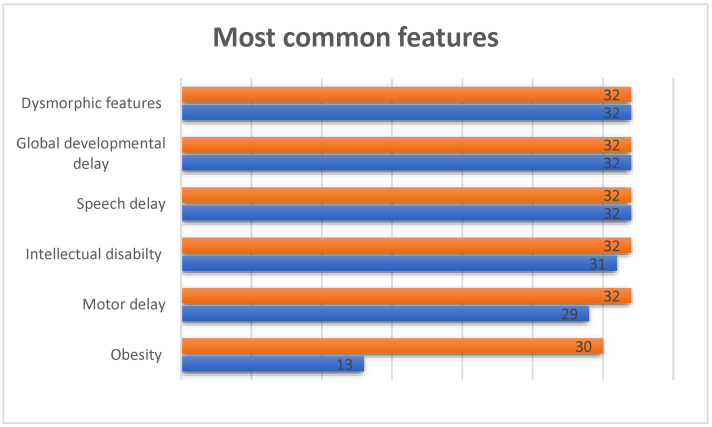
Most common features in individuals with TRIP12 variants. The orange bar shows the number of individuals in our cohort for whom phenotypic information was available and the blue bar shows how many individuals in our cohort scored positive on this particular clinical feature.

Clark–Baraitser syndrome is associated with dysmorphic features, intellectual disability accompanied by autism spectrum disorder, speech delay and obesity. It can also lead to motor and global developmental delay (GDD). In our cohort, all individuals have dysmorphic features, speech delay and global developmental delay. Figure 2 shows the most common features observed in the cohorts. Individual 22 was the only case without evidence of intellectual disability. Individuals 9, 26 and 27 did not show signs of motor delay, and obesity was observed in 13 individuals (1, 2, 6–8, 12, 14–15, 21, 23, 26, 30 and 32). It is possible, however, that some features were not detected during examination because of the young age of the individuals.

### 2.2. TRIP12 Episignature Shows an Overall Decrease in DNA Methylation

**Figure 3:**
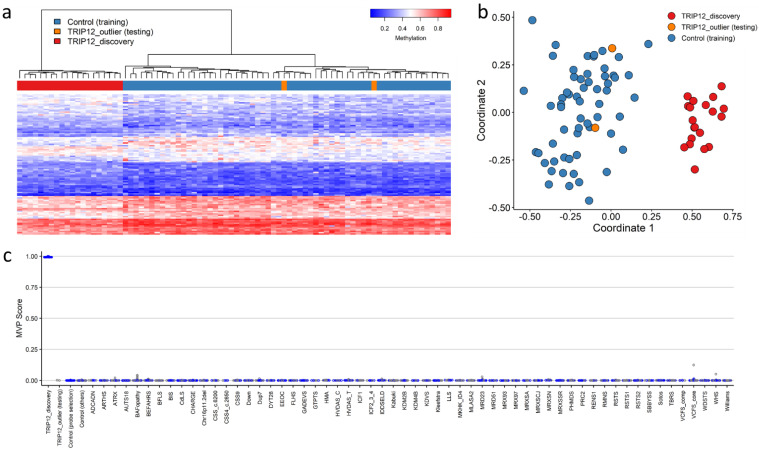
Clark–Baraitser syndrome (TRIP12) episignature—discovery cohort: (**a**) Euclidean hierarchical clustering (heatmap): each column represents a single TRIP12 discovery case or control; each row represents 1 of the 105 CpG probes selected for the episignature. This heatmap shows clear separation between 20 TRIP12 cases (red) from controls (blue). Two outlier cases (orange) are shown to segregate with controls; (**b**) Multidimensional scaling (MDS) plot shows segregation of TRIP12 cases from both controls and outlier cases; (**c**) Support Vector Machine (SVM) classifier model. The model was trained using the 105 selected TRIP12 episignature probes, 75% of controls and 75% of other neurodevelopmental disorder samples (blue). The remaining 25% controls and 25% of other disorder samples were used for testing (grey). Plot shows the TRIP12 discovery cases with a methylation variant pathogenicity (MVP) score close to 1 compared with all other samples, showing the specificity of the classifier and episignature.

We sought to determine whether variants in *TRIP12* would cause a detectable change in DNA methylation. We compared methylation beta values between cases with confirmed variants in *TRIP12* and controls. Two discovery cases (Cases 21 and 22) were shown not to map to an episignature and were subsequently not included as training samples for episignature discovery and labeled as outliers. These were plotted alongside the discovery episignature (labeled TRIP12_outlier, Figure 3). Using the remaining cohort of 20 discovery cases, 105 differentially methylated CpG probes were retained for the *TRIP12* discovery episignature (mean methylation difference: 5–13%; adjusted *p*-value: 0.001 to 0.44) (Supplementary Table S1). Hierarchical clustering (heatmap) demonstrated that the selected CpG probes were capable of segregating the *TRIP12* cases from age- and sex-matched controls (Figure 3a). Subsequently, unsupervised multidimensional scaling (MDS) showed a clear separation between discovery cases and controls and confirmed the robustness of the episignature (Figure 3b). Leave-25%-out cross validation was performed using unsupervised hierarchical and MDS clustering methods and confirmed the robustness and sensitivity of the episignature. All testing cases were correctly clustered with the discovery training cases (Supplementary Figure S1).

Finally, we constructed a support vector machine classifier (SVM) model using the 105 selected discovery episignature probes. All training cases showed an MVP score close to 1, indicating the similarity of the observed methylation pattern to the *TRIP12* episignature (Figure 3c).

### 2.3. Validation of the TRIP12 Episignature

**Figure 4:**
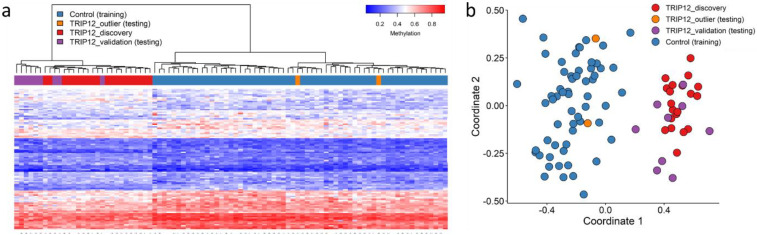
Validation of the Clark–Baraitser syndrome (TRIP12) episignature—validation cohort: (**a**) Euclidean hierarchical clustering (heatmap): each column represents a single TRIP12 case or control; each row represents 1 of the 105 CpG probes selected for the episignature. This heatmap shows segregation of the 9 TRIP12 validation cases (purple) with the 20 TRIP12 training (discovery) cases (red) from controls (blue). The 2 outlier cases (orange) remain segregated with controls; (**b**) Multidimensional scaling (MDS) plot shows segregation of TRIP12 cases (validation and discovery) from controls.

In order to validate the *TRIP12* episignature, we assessed nine additional cases with confirmed pathogenic variants in *TRIP12* (Cases 24–32; Table 1). We performed hierarchical clustering and MDS and confirmed that all validation cases clustered with training cases (Figure 4).

### 2.4. The TRIP12 Episignature Can Be Used to Classify Variants of Uncertain Significance (VUS)

To obtain a more robust episignature, after confirming that the *TRIP12* episignature could correctly identify the validation samples, we added the nine validation samples to the training (discovery) cohort and repeated probe selection. We obtained a final list of 118 differentially methylated probes (mean methylation difference: 5–15%; adjusted p-value: 1.15 × 10^−7^ to 0.002) (Supplementary Table S2). Leave-25%-out cross validation was repeated and all testing cases clustered with training cases (Supplementary Figure S2).

**Figure 5:**
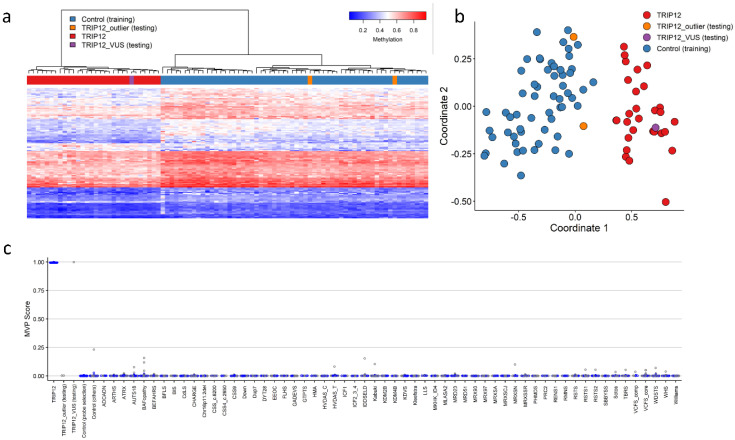
Assessment of *TRIP12* variant of uncertain significance (VUS) using the Clark–Baraitser syndrome (*TRIP12*) episignature: (**a**) Euclidean hierarchical clustering (heatmap): each column represents a *TRIP12* case or control; each row represents 1 of the 118 CpG probes selected for the episignature. This heatmap shows clear separation between 29 *TRIP12* cases (red) used for training from controls (blue). The VUS case (purple) is shown to segregate with training cases. Two outlier cases (orange) are shown to segregate with controls; (**b**) Multidimensional scaling (MDS) plot shows the segregation of the *TRIP12* VUS case with training and away from both controls and outlier cases; (**c**) Support Vector Machine (SVM) classifier model. Model was trained using the 118 selected *TRIP12* episignature probes, 75% of controls and 75% of other neurodevelopmental disorder samples (blue). The remaining 25% controls and 25% of other disorder samples were used for testing (grey). Plot shows the *TRIP12* VUS case with a methylation variant pathogenicity (MVP) score close to 1, similar to the *TRIP12* training cases, showing the specificity of the classifier and episignature.

Episignatures have been shown to be capable of aiding in the classification of variants of uncertain significance [21]. Therefore, we applied this *TRIP12* episignature to a sample with a VUS (Case 23, Table 1) and classified the sample using unsupervised (MDS and hierarchical) clustering and supervised (SVM) methods. The VUS sample clustered with the *TRIP12* training samples in MDS and hierarchical clustering (Figure 5) and had a prediction score close to 1 (Figure 5c). We observed that the VUS can be reclassified as likely pathogenic or pathogenic.

### 2.5. Overlap of the TRIP12 Genome-Wide DNA Methylation Profile with Other Neurodevelopmental Disorders Conditions on EpiSign™

**Figure 6:**
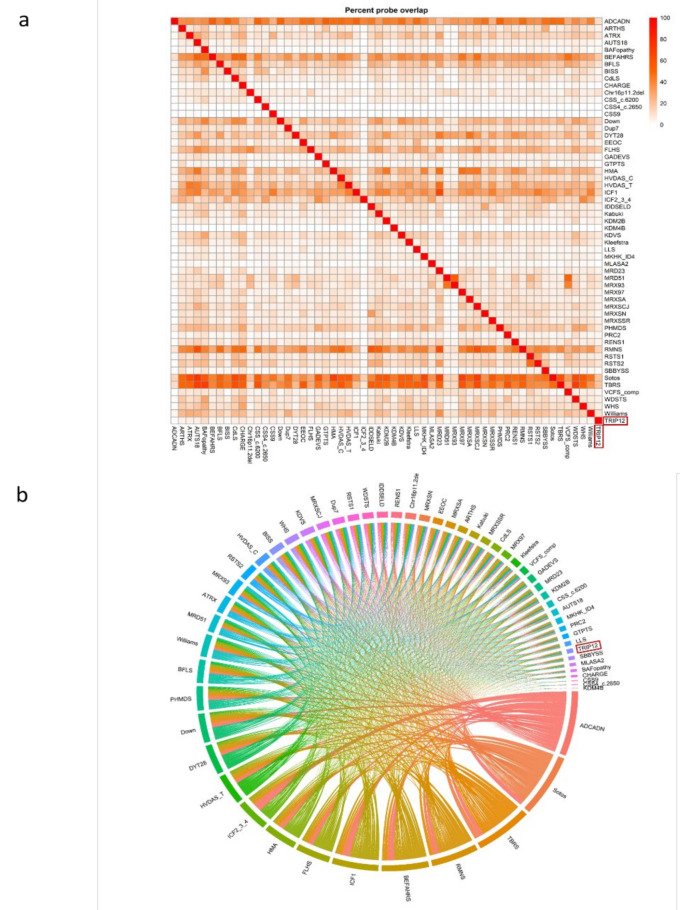
Differentially methylated probes (DMPs) shared between the *TRIP12* cohort and 56 other EpiSign™ disorders with known episignatures: (**a**) heatmap showing the percentage of probes shared between each paired cohort. Colors indicate the percentage of the y-axis cohort’s probes that are also found in the x-axis cohort’s probes. (**b**) Circos plot representing the probes shared between each pair of cohorts. The thickness of the connecting lines indicates the number of probes shared between the two cohorts. Abbreviations are listed in Supplementary Table S3.

To perform analysis of differentially methylated regions (DMRs) and functional annotation, we again compared the *TRIP12* cohort to age-, sex- and array-matched controls from the EpiSign™ Knowledge Database (EKD). Only controls without a known episignature or controls unaffected by a neurodevelopmental phenotype were used in this analysis. We generated a list of differentially methylated probes (DMPs) for the *TRIP12* cohort and compared it to the DMP lists for 56 other episignature cohorts on the EpiSign™ v3 clinical classifier previously described by Levy et al. [25] (Figure 6). A list of EpiSign™ disorders and their abbreviations are listed in Supplementary Table S3. There were 4813 DMPs for *TRIP12*, and the range across all cohorts was 279 to 151,848 DMPs [25]. The highest percent overlap of the *TRIP12* DMPs with other EpiSign™ disorders were for BAFopathy (~10%, including *ARID1A*, *ARID1B*, *SMARCB1*, *SMARCA2*, *SMARCA4*), CHARGE (~8%, *CHD7*) and MRD23 (~8%, *SETD5*) (Figure 6a).

**Figure 7:**
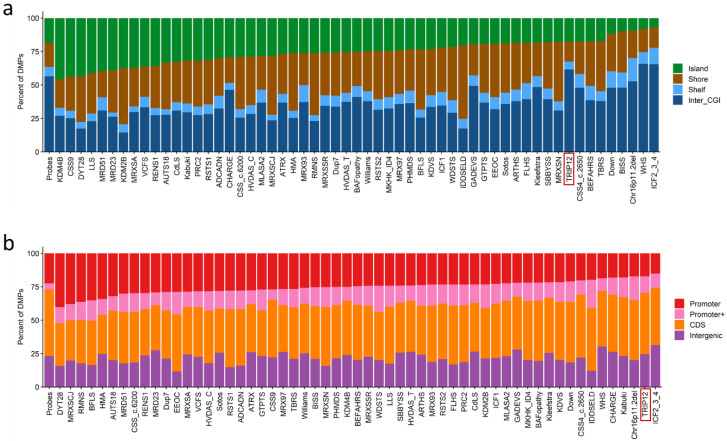
Differentially methylated probes (DMPs) annotated in the context of CpG islands and genes: (**a**) DMPs annotated in the context of CpG islands. Island, CpG islands; Shore, within 0–2 kb of a CpG island boundary; Shelf, within 2–4 kb of a CpG island boundary; Inter_CGI, all other regions in the genome. (**b**) DMPs annotated in the context of genes. Promoter, 0–1 kb upstream of the transcription start site (TSS); Promoter+, 1–5 kb upstream of the TSS; CDS, coding sequence; Intergenic, all other regions of the genome. The Probes column in both (**a**,**b**) represents the background distribution determined in the Levy et al. study [25] of all array probes after initial filtering and used as input for DMP analysis. Abbreviations of all array probes after initial filtering and used as input for DMP analysis are listed in Supplementary Table S3 [25].

Next, we annotated the genomic location of the DMPs, e.g., in relation to CpG islands and genes. This showed that the DMPs are predominantly found in genomic regions outside of CpG islands and the shore/shelf regions (Figure 7a). Similarly, when annotated in relation to genes, the DMPs are predominantly found in coding regions or other intergenic regions and not promoter regions. (Figure 7b).

**Figure 8:**
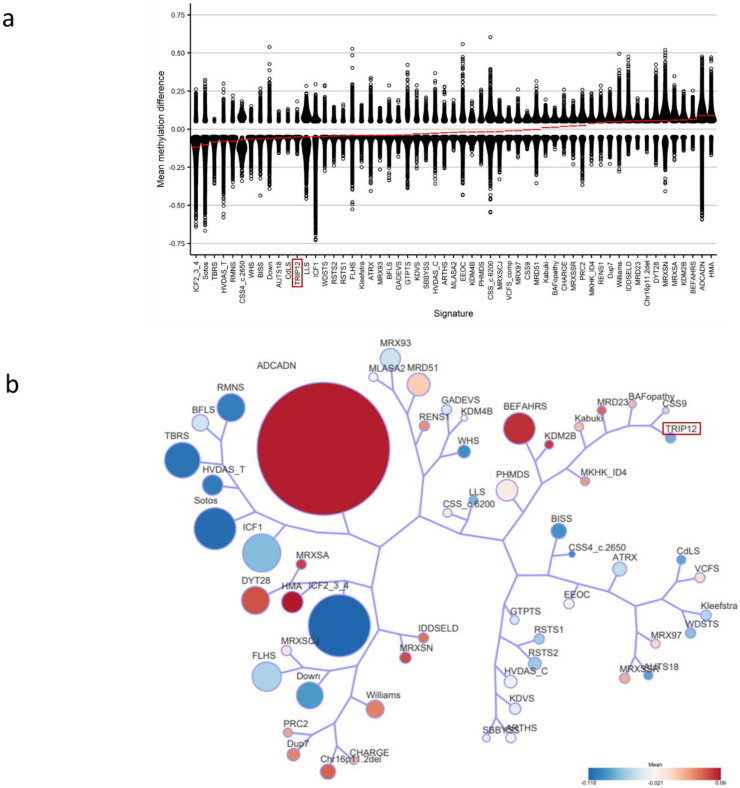
Relationships between the *TRIP12* cohort and 56 other EpiSign™ disorders: (**a**) Methylation differences of all differentially methylated probes (DMPs) for each cohort, sorted by mean methylation. Each circle represents one probe. Red lines indicate mean methylation; (**b**) Tree and leaf visualization of Euclidean clustering of all 57 cohorts using the top n DMPs for each group, where n = min (# of DMPs, 500). Cohort samples were aggregated using the median value of each probe within a group. A leaf node represents a cohort, with node sizes illustrating relative scales of the number of selected DMPs for the corresponding cohort, and node colors are indicative of the global mean methylation difference. Abbreviations are listed in Supplementary Table S3.

Lastly, we assessed the relationship between the *TRIP12* cohort and the 56 other EpiSign™ disorders (Figure 8). All DMPs were used to calculate the mean beta-values for each cohort and determine the overall methylation trend, i.e., hypo- or hypermethylation. *TRIP12* had a mean methylation indicating predominantly hypomethylation changes (Figure 8a). Clustering analysis was then performed using the top 500 DMPs for each cohort to assess similarity in genome-wide methylation profiles. For cohorts with less than 500 DMPs, the total DMPs for those cohorts were used in analysis. This analysis showed that *TRIP12* is most closely related to the CSS9 (*SOX11*), BAFopathy (*ARID1A*, *ARID1B*, *SMARCB1*, *SMARCA2*, *SMARCA4*) and MRD23 (*SETD5*) episignatures (Figure 8b).

### 2.6. Differentially Methylated Regions (DMRS)

Using the DMRcate algorithm [26] with p-cutoff set to default (FDR) and a beta-cutoff input of 5% mean methylation difference and 5 CpGs, we identified 36 DMRs (Supplementary Table S4). All 36 of these were hypomethylation events, 30 of which were in gene bodies and 6 were intergenic regions. This is in line with the genomic regions of the DMPs identified in Figure 7. In particular, three DMRs involve genes with a similar or overlapping clinical phenotype and all three regions lie within the gene body. The first DMR involves *STAT3* (OMIM #102582) at chromosome region 17q21.2, which is associated with the autosomal dominant condition hyper-IgE recurrent infection syndrome (HIES1 OMIM #147060). HIES1 results in facial features similar to those reported in Clark–Baraitser syndrome including prominent forehead and hypertelorism. The second region involves *PBX1* (OMIM #176310) at chromosome region 1q23.3, which is also associated with an autosomal dominant neurodevelopmental disorder: congenital anomalies of kidney and urinary tract syndrome with or without hearing loss, abnormal ears or developmental delay (OMIM #617641). This disorder has several overlapping clinical features with Clark–Baraitser syndrome including facial dysmorphisms of the philtrum and ear lobes, epicanthal folds and strabismus as well as hypotonia, developmental and speech delay. The third region involves *TRAPPC9* (OMIM #611966) at chromosome region 8q24.3 associated with autosomal recessive intellectual developmental disorder 13 (OMIM #613192). Overlapping phenotypic features include truncal obesity, hypertelorism, intellectual disability, delayed speech, seizures and hyperactivity.

## 3. Discussion

DNA methylation patterns are established during embryonic development and are detectable in peripheral blood as easily accessible diagnostic biomarkers [16]. DNA methylation analysis has been used to identify specific episignatures associated with a growing number of Mendelian neurodevelopmental disorders. Episignature analysis can enable a definitive molecular diagnosis for unsolved cases of ID and reclassify VUSs found by WES or large NGS panel analysis [22].

The aim of this study was to detect and validate a DNA methylation episignature for *TRIP12* aberrations and to gain more knowledge about the underlying molecular pathway of *TRIP12* aberrations based on the affected loci in the broader genome-wide DNA methylation profile. In this study, we describe a specific DNA methylation episignature for pathogenic *TRIP12* aberrations. DNA methylation profiles were collected from peripheral blood of a cohort of 32 individuals with confirmed variants in *TRIP12*. The classification model for *TRIP12* was built with a discovery cohort (20 individuals with (likely) pathogenic variants) and a control set (60 matched control samples from the EKD) using 105 DMPs. Hierarchical clustering and MDS visualization showed a clear distinction between individuals with (likely) pathogenic variants and controls, indicating robustness of the *TRIP12* episignature. In order to assess the reproducibility, we performed leave-25%-out cross validation. During the cross validation, all of the testing cases clustered together with training cases, which confirmed the reproducibility of the episignature. Our SVM classification model showed that the episignature was highly specific and sensitive, and confirmed that *TRIP12* biomarkers could be differentiated from other EpiSign™ disorders associated with developmental delay and intellectual disability.

However, two individuals (Cases 21 and 22), whom we believed to have (likely) pathogenic variants, clustered with controls, indicating the absence of the *TRIP12* episignature. Case 21 with the c.2482C>G p.(Pro828Ala) variant had obesity and mild intellectual disability, features which are not unique for *TRIP12* variants. Overall, in silico tools predicted this missense variant to be tolerated (SIFT [27]: Tolerated, score 0.33; PolyPhen-2 [28]: Probably Damaging, score 0.998; Align GVGD [29]: class C0; and Grantham [30]: score 27). Case 22 with the c.5800 C>A p.(Pro1934Thr) variant was born prematurely and had abnormal facial shape, delayed speech, language, and gross motor development. These characteristics are in line with the expected syndrome phenotype of a *TRIP12* variant. Intellectual disability can, by definition, not reliably be diagnosed before 3 years of age. This individual underwent WES and WGS, but no other possible genetic mutation was found to explain the phenotype. Overall, in silico predictions indicated this variant to be equivocal (SIFT [27]: Deleterious, score 0; PolyPhen-2 [28]: Possibly Damaging, score 0.503; Align GVGD [29]: class C35; and Grantham [30]: score 38). The absence of this episignature may indicate that these variants do not result in loss of the *TRIP12* function. No other possible disease-causing variants were identified by WES and WGS in these two cases. Additionally, a chromosomal microarray was not performed in either of these cases and, therefore, we cannot rule out the presence of a copy number variant that could be contributing to the reported phenotypes. Further research is needed for these variants.

After confirming that the *TRIP12* episignature correctly classified the validation set, we added the nine validation samples to the training cohort and repeated the analysis, in order to further refine the episignature, resulting in 118 episignature probes.

Overlap in clinical features in genetic disorders complicates and can create ambiguity in clinical diagnosis, requiring a confirmatory genetic diagnosis. Limitations and ambiguities in genetic testing, including VUSs, often result in inconclusive findings and uncertain clinical diagnosis. Use of episignature analysis has been demonstrated to increase the diagnostic yield and improve our ability to interpret ambiguous genetic findings. Accordingly, DNA methylation testing has been proposed and implemented as a novel molecular diagnostic test [22,23]. We tested an individual with a *TRIP12* VUS (Case 23) to determine if it mapped to the refined *TRIP12* episignature. The VUS clustered together with the *TRIP12* episignature samples in the MDS plot and yielded an MVP score near one, clearly showing the presence of the *TRIP12* episignature. These findings indicate that this individual carried a genetic variant that results in disruption of *TRIP12* function and, therefore, the variant can be reclassified as likely pathogenic or pathogenic. The results of our study reinforce the clinical use of episignatures as biomarkers by defining a novel and specific DNA methylation episignature that can be utilized as a diagnostic tool to resolve VUSs in the *TRIP12* gene.

Our newly discovered *TRIP12* signature was trained against 56 known signatures, indicating sufficient selectivity and sensitivity. Although these other 56 syndromes may share phenotypic characteristics with Clark–Baraitser syndrome, we can, at this point, not exclude the existence of additional or nested signatures in relation to inconclusive results. Such signatures may represent a particular subset of patients, i.e., carrying mutations for a particular functional gene domain or mutations in different genes that involve the same functional active protein complex. It should be noted that in our workflow, updates on variant classification databases and screening of inconclusive/unresolved patient signatures within newly discovered signatures are standard operating procedures within the signature discovery workflow of our laboratory [11,21].

We showed the strong effect of genetic variation within *TRIP12* on DNA methylation, resulting in predominant genomic hypomethylation. Our results demonstrated that all of the differentially methylated regions identified in individuals with a *TRIP12* (likely) pathogenic variant were hypomethylated. Therefore, we can hypothesize that functional proteins within this pathway might lead directly to hypomethylation of specific loci.

Thirty-six hypomethylated DMRs were identified including those involving intragenic regions of *STAT3*, *PBX1* and *TRAPPC9*. These genes are associated with disorders with overlapping clinical features to Clark–Baraitser syndrome including intellectual disability, hypotonia, developmental delay, speech delay and facial dysmorphisms. The presence of DMRs in gene bodies and intergenic regions demonstrates that there are genome-wide DNA methylation changes. Additional research, including gene expression profiling, will be useful to correlate with the functional impact of these epigenetic changes.

We demonstrated that the differentially methylated probes (DMPs) that were identified in *TRIP12* overlapped with that of BAFopathies (~10%). BAFopathies describe several neurodevelopmental disorders that are caused by disruption to genes within the BRG1/BRM-associated factor (BAF) complex [11]. BAF is a chromatin remodeling complex that plays a major role in the regulation of gene expression and differentiation [31]. Interestingly, *TRIP12* interacts with *SMARCE1* (BAF57), a subunit of the BAF Switch/Sucrose Non-Fermentable (SWI/SNF) chromatin remodeling complex and has been postulated to act as a quality control system of BAF57 or the entire SWI/SNF complex [5,10]. Therefore, aberrations of *TRIP12* could result in disruption of proper chromatin structure, the consequences of which would include aberrant DNA methylation, as described here, similar to that seen in other BAF complex genes [11]. We have shown that when we assess just over 100 of the most differentiating probes, the EpiSign™ classifier can discern *TRIP12* from 56 other neurodevelopmental disorder episignatures including those associated with BAFopathies. The current EpiSign™ BAFopathy episignature encompasses several subtypes of Coffin–Siris syndrome associated with multiple genes (*ARID1A*, *ARID1B*, *SMARCB1*, *SMARCA4*) and Nicolaides–Baraitser syndrome (*SMARCA2*) [21]. Further work is ongoing to determine if *SMARCE1* will also show DNA methylation changes similar to the other BAF complex genes. In addition, this work permits assessment of the potential overlap with the *TRIP12* genome-wide DNA methylation profile and gives further insight into the pathogenesis of Clark–Baraitser syndrome. We also demonstrate that *TRIP12* is closely related to the Coffin–Siris syndrome 9 (CSS9) episignature, which is the result of variants in *SOX11*. *SOX11* is a transcription factor involved in the downstream pathways of the BAF complex thought to play a crucial role in brain development [32]. SOX11 is shown to initiate chromatin opening and creates a permissive state to initiate transcription and thereby leads to changes in gene expression [33]. Taken together, our findings support the interconnectivity of the roles of *TRIP12*, *SMARCE1* and *SOX11* and confirms the *TRIP12* episignature as a specific biomarker that can be utilized for the diagnosis of Clark–Baraitser syndrome. We postulate that further work on assessing the epigenetic consequences of other *TRIP12*-interacting genes may provide evidence of additional episignatures.

## 4. Materials and Methods

### 4.1. Subjects and Study Cohort

In this project, we included a total of 32 individuals, of which 28 have been previously described clinically (Cases 1–3, 5–9, 12–21, 23, 24 and 26–32) [24]. The 32 individuals were divided into two different cohorts: one cohort for the discovery of the episignature (n = 22) and one cohort to validate the episignature and assess VUS (n = 10). The discovery cohort was used for the purpose of episignature probe selection and construction of the classification model. The *TRIP12* variants were identified through single WES (whole-exome sequencing), trio WES, Sanger sequencing, gene panels, array CGH and WGS (whole-genome sequencing) and were classified according to the guidelines of the American College of Medical Genetics (ACMG) [34].

The discovery cohort involved a total of 22 individuals (15 females and 7 males) with confirmed pathogenic or likely pathogenic variants in *TRIP12*, 2 deletions of at least one exon of *TRIP12*, 8 frameshift, 5 nonsense, 3 splice site and 4 missense variants.

The validation cohort involved a total of 10 individuals (5 females and 5 males) with variants in *TRIP12*. Nine individuals carried a pathogenic or likely pathogenic variant, including 1 deletion of at least one exon of *TRIP12*, 5 frameshift, 2 nonsense and 1 splice site variants. The established episignature was used to assess pathogenicity of 1 individual carrying a VUS as part of the validation cohort. This individual carried a VUS involving an in-frame deletion.

Controls were randomly selected from the EKD, [35] matched for age, sex and array type.

### 4.2. Sample Processing

DNA was obtained from whole blood using standard techniques. Bisulphite conversion was performed according to Illumina protocol (Illumina, San Diego, CA, USA). DNA methylation analysis was carried out using 500 ng of bisulfite-converted DNA as the input for the Illumina Infinium MethylationEPIC BeadChip arrays (San Diego, CA, USA) according to manufacturer’s protocols. In order to minimize batch effect, samples were randomly divided over separate batches.

Analysis and discovery of episignatures were carried out based on our laboratory’s previously published protocols [22,23,35]. In brief, intensity data files (IDATs) containing methylated and unmethylated signal intensities were analyzed in R (version 4.1.1). The methylation data normalization was performed using the Illumina normalization method with background correction using the minfi package (version 1.40.0) (accessed on 2 July 2022) [36]. From the 866,836 total probes available, the following probes were eliminated; probes with detection *p* value > 0.1, probes located on chromosomes X and Y (n = 19,120 and n = 561, respectively), probes containing single-nucleotide polymorphisms (SNPs) at or near the CpG interrogation site or single-nucleotide extension sites (n = 31,647) and probes that cross-react with other genomic regions (n = 72,487). Samples containing failed probes of more than 5% (*p*-value > 0.1, calculated by the minfi package) were removed. Principal component analysis (PCA) was performed to examine batch structure and identify case or control outliers. Matched controls were randomly selected from the EKD [22] and matched by age, sex and array type using the MatchIt package (version 4.3.4) [37] at a ratio of 1:5. Methylation levels for each probe (beta values) were converted to M-values by logit transformation and linear regression applied to identify differentially methylated probes using the limma package (v3.50.0) [38]. Estimated blood cell proportions were incorporated into the model matrix as confounding variables [39]. The blood cell types used as covariates are CD4+ and CD8+ cells, natural killer cells, monocytes, granulocytes and B-cells as described in the minfi package. p-values were moderated using the eBayes function in the limma package.

### 4.3. Probe Selection and Episignature Classifier Construction

Selection of probes for the episignatures were performed in three steps. Firstly 900 to 1000 probes were retained with the highest product of absolute methylation differences between cases and controls and the negative of the logarithm of *p*-values. Secondly, a receiver’s operating characteristic (ROC) curve analysis was performed, and 300 to 500 probes were retained with the highest area under the ROC curve (AUC). Lastly, probes with pair-wise correlation greater than 0.60 measured using Pearson’s correlation coefficients for all probes were eliminated. Unsupervised clustering models were applied using the remaining probes, including hierarchical clustering (heatmap) using Ward’s method on Euclidean distance in the gplots package in R (v3.1.1) and multidimensional scaling (MDS) by scaling of the pair-wise Euclidean distances between samples. To assess the robustness of the episignatures, multiple rounds of leave-25%-out cross validation were performed. In each round, 25% of *TRIP12* samples were used as testing samples and the remaining samples were used for probe selection. The corresponding unsupervised clustering plots were visualized. The e1071 R package (version 1.7-9) was used to train a support vector machine (SVM) classifier and construct a multi-class prediction model as previously described [21].

### 4.4. Functional Annotation and Comparison between EpiSign™ Cohort

Functional annotation and EpiSign™ cohort comparisons were performed according to our previously published methods [25]. In short, to assess the percentage of differentially methylated probes (DMPs) shared between the *TRIP12* episignature and the 56 other neurodevelopmental conditions on the EpiSign™ clinical classifier, heatmaps and circos plots were produced. Heatmaps were plotted using the R package pheatmap (version 1.0.12) and circos plots using the R package circlize (version 0.4.15) [40]. To determine the genomic location of the DMPs, probes were annotated in relation to CpG islands (CGIs) and genes using the R package annotatr (version 1.20.0) [41] with AnnotationHub (version 3.2.2) and annotations hg19_cpgs, hg19_basicgenes, hg19_genes_intergenic, and hg19_genes_intronexonboundaries. CGI annotations included CGI shores from 0–2 kb on either side of CGIs, CGI shelves from 2–4 kb on either side of CGIs, and inter-CGI regions encompassing all remaining regions. For gene annotations, promoters included the region up to 1Kb upstream of the transcription start site (TSS) and promoter+ included the region 1–5 Kb upstream of the TSS. Annotations to untranslated regions (5′-UTR and 3′ UTR), exons, introns and exon/intron boundaries were combined into the “gene body” category. In order to assess the relationship between the *TRIP12* cohort and the 56 other EpiSign™ disorders, the distance and similarities between cohorts were analyzed using clustering methods and visualized on a tree and leaf plot. This assessed the top 500 DMPs for each cohort, ranked by p-value. For cohorts with less than 500 DMPs, all DMPs were used. Tree and leaf plots were generated using the R package TreeAndLeaf (version 1.6.1), showing additional information including global mean methylation difference and total number of DMPs identified for each cohort.

### 4.5. Differentially Methylated Regions (DMRs)

Differentially methylated regions (DMRs) were detected using the DMRcate package in R (v 2.8.3) [26], and regions containing at least 5 significantly different CpGs within 1kb, with a minimum mean methylation difference of 5% and a Fisher’s multiple comparison *p*-value < 0.01, were considered significant. DMRs were annotated using the UCSC Genome Browser Data Integrator with GENCODE V3lift37 comprehensive annotations and further characterized using UCSC Genome Browser tools (accessed on 3 March 2022) (https://genome.ucsc.edu).

## 5. Conclusions

In this study, we identified a highly specific DNA methylation episignature for individuals with a (likely) pathogenic variant in *TRIP12*. We recommend that this episignature be used to assess and reclassify *TRIP12* variants. This episignature will be added to the diagnostic tool EpiSign™.

## Figures and Tables

**Table 1:** Molecular details of the cohorts.

ID	Sex	Age	Variants	Origin	Diagnostic Test	Variant	Cohort Type
1	F	49	c.1921C>T (p.Gln641*)	not intherited from mother	gene panel	nonsense	discovery
2	F	28	c.5293del p.(Asp1765llefs*13)	de novo	WES	frameshift	discovery
3	M	8	c.2612del p.(Ala871Valfs*4)	de novo	WES	frameshift	discovery
4	F	20	c.2426-1G>A	NA	NA	splice site	discovery
5	F	12	c.2773+1G>A	de novo	gene panel	splice site	discovery
6	F	19	2q36.3(229824569_230111952) × 1 Deletion exons 1–7	de novo	array CGH	deletion	discovery
7	M	9	2q36.3(229796589_229802461) × 1 Deletion exons 20–24	not intherited from mother	WES	deletion	discovery
8	F	13	c.5576C>G p.(Pro1859Arg)	de novo	NA	missense	discovery
9	F	9	c.4628del p.(Pro1543Leufs*11)	de novo	WES	frameshift	discovery
10	F	3	c.4068_4069dup p.Met1357Thrfs*2	de novo	trio WES	frameshift	discovery
11	M	10	c.2771A>T p.(Glu924Val)	de novo	trio WES	missense	discovery
12	F	17	c.1192G>T p.(Glu398*)	de novo	gene panel	nonsense	discovery
13	F	21	c.1507C>T p.(Arg503*)	de novo	WES	nonsense	discovery
14	M	10	c.1507C>T p.(Arg503*)	de novo	gene panel	nonsense	discovery
15	F	4	c.2361_2362del p.(Asn787Lysfs*14)	de novo	trio WES	frameshift	discovery
16	F	12	c.3361C>T p.(Gln1121*)	de novo	gene panel	nonsense	discovery
17	M	7	c.3583del p.(Ser1195Leufs*24)	de novo	gene panel	frameshift	discovery
18	F	21	c.3828_3829del p.(Ala1277Lysfs*13)	de novo	NA	frameshift	discovery
19	M	65	c.5411dup p.(Lys1805Glufs*28)	NA	WES	frameshift	discovery
20	F	22	c.5583+1G>A	de novo	gene panel	splice site	discovery
21	F	66	c.2482C>G p.(Pro828Ala)	de novo	WES	missense	discovery_outlier
22	M	2	c.5800 C>A p.Pro1934Thr	de novo	WES	missense	discovery_outlier
23	F	8	c.1503_1511del p.Cys502_Ala504del)	de novo	WGS	in-frame	validation_VUS
24	M	11	c.3743+1G>A	de novo	trio WGS	splice site	validation
25	F	14	c.1025_1026ins44 p. (Ser343Cysfs*17)	NA	NA	frameshift	validation
26	F	7	c.1983delC p.(Ile662Phefs*2)	de novo	WES	frameshift	validation
27	F	8	c.1896_1911+30del p.(Asn632Lysfs*21)	de novo	trio WES	frameshift	validation
28	M	13	2q36.3(229810880_229836984) × 1 Deletion exons 5–14	de novo	trio WES, array CGH	deletion	validation
29	M	7	c.3370del p.(Cys1124Valfs*19)	de novo	WES	frameshift	validation
30	M	21	c.1132C>T p.(Gln378*)	de novo	WES	nonsense	validation
31	M	3	c.1132C>T p.(Gln378*)	inherited from affected father	Sanger sequencing	nonsense	validation
32	F	18	c.1195dup p.(Met399Asnfs*31)	de novo	trio WGS	frameshift	validation

Variants are based on NM_004238.3. NA; not assessed. Cases 13 and 14 are not related. Cases 30 and 31 are related.

## Data Availability

The raw DNA methylation data for the samples are not available due to institutional and ethics restrictions.

## Supplementary Materials

The supporting information can be downloaded at: https://www.mdpi.com/article/10.3390/ijms232213664/s1.

## Abbreviations

*TRIP12*, Thyroid Hormone Receptor Interactor 12; ID, Intellectual disability; ASD, autism spectrum disorder; HECT, Homologous to the E6-AP Carboxyl Terminus; WWE, Tryptophan–Tryptophan–Glutamate; ARM, Armadillo repeats; IDR, Intrinsically Disordered Regions; VUS, variant of unknown significance; ACMG, American College of Medical Genetics; EKD, EpiSign Knowledge Database; SNPs, single-nucleotide polymorphisms; PCA, principal component analysis; AUROC, areas under the receiver operating characteristic curve; MDS, multidimensional scaling; SVM, support vector machine; DMR, differentially methylated region; DMP, differentially methylated probes; WES, whole-exome sequencing; WGS, whole-genome sequencing.
