# Supplementary material for: Episignature Mapping of TRIP12 Provides Functional Insight into Clark–Baraitser Syndrome
Source: Int J Mol Sci. 2022 Nov 8;23(22):13664. doi: 10.3390/ijms232213664 (PMC9690904; doi:10.3390/ijms232213664)

Supplementary Figure 1: Leave-25%-out cross validation (Discovery Cohort)

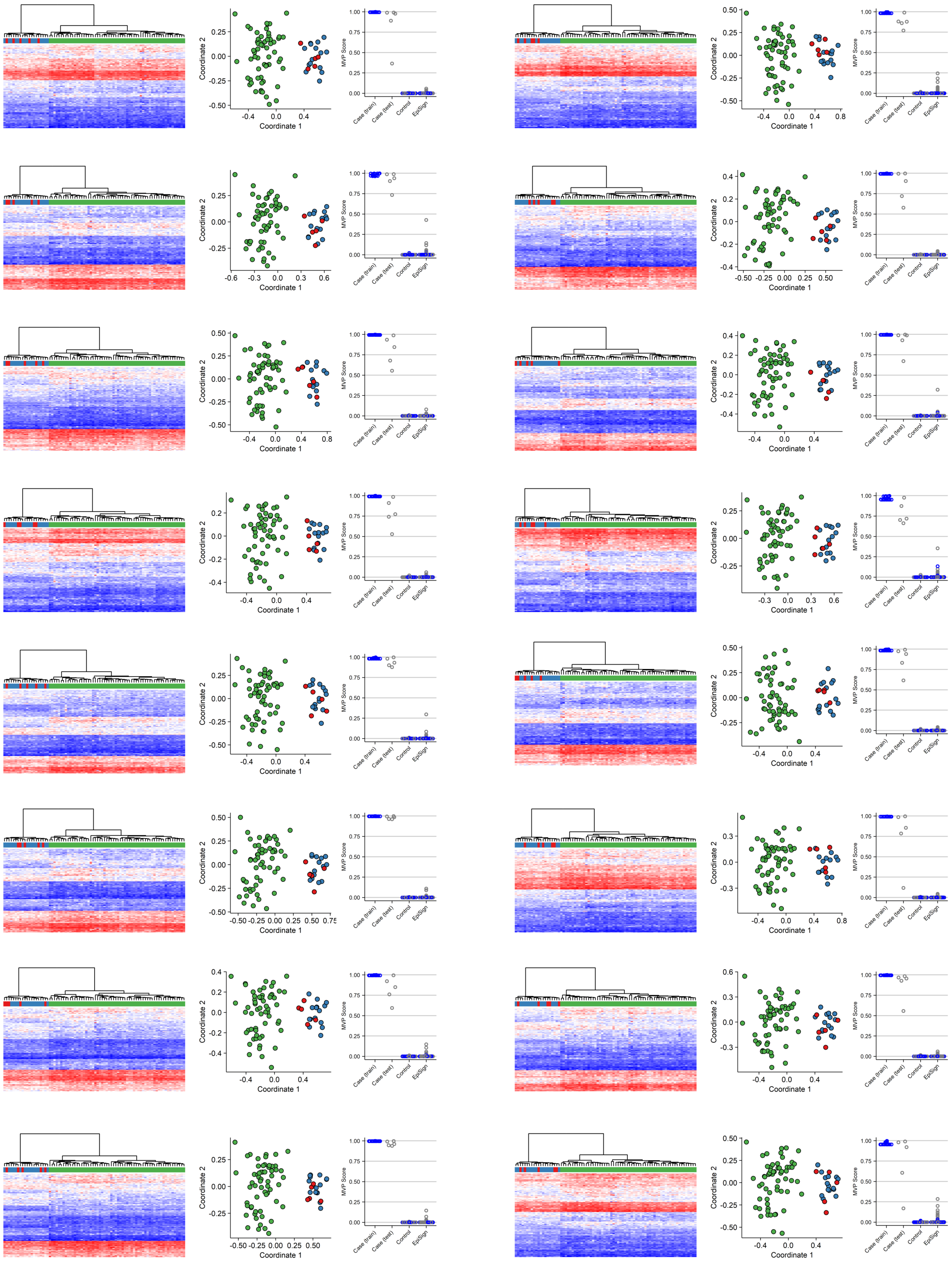

Supplementary Figure 1: Leave-25%-out cross validation (Discovery Cohort)

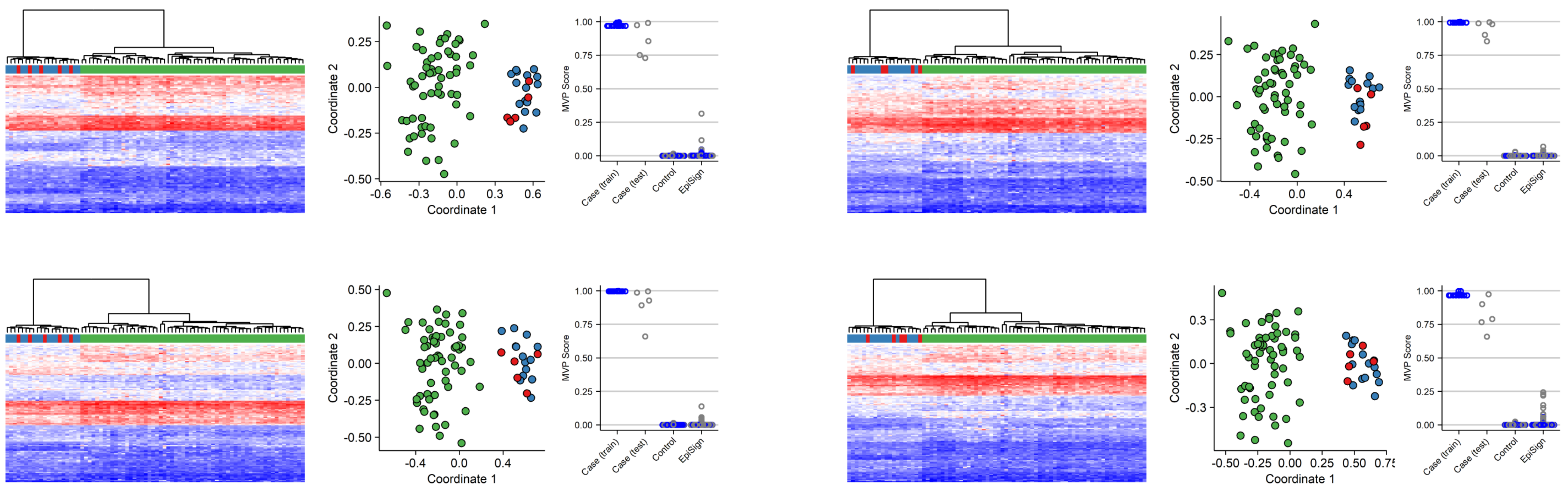

Supplementary Figure 2: Leave-25%-out cross validation (Discovery + validation Cohort)

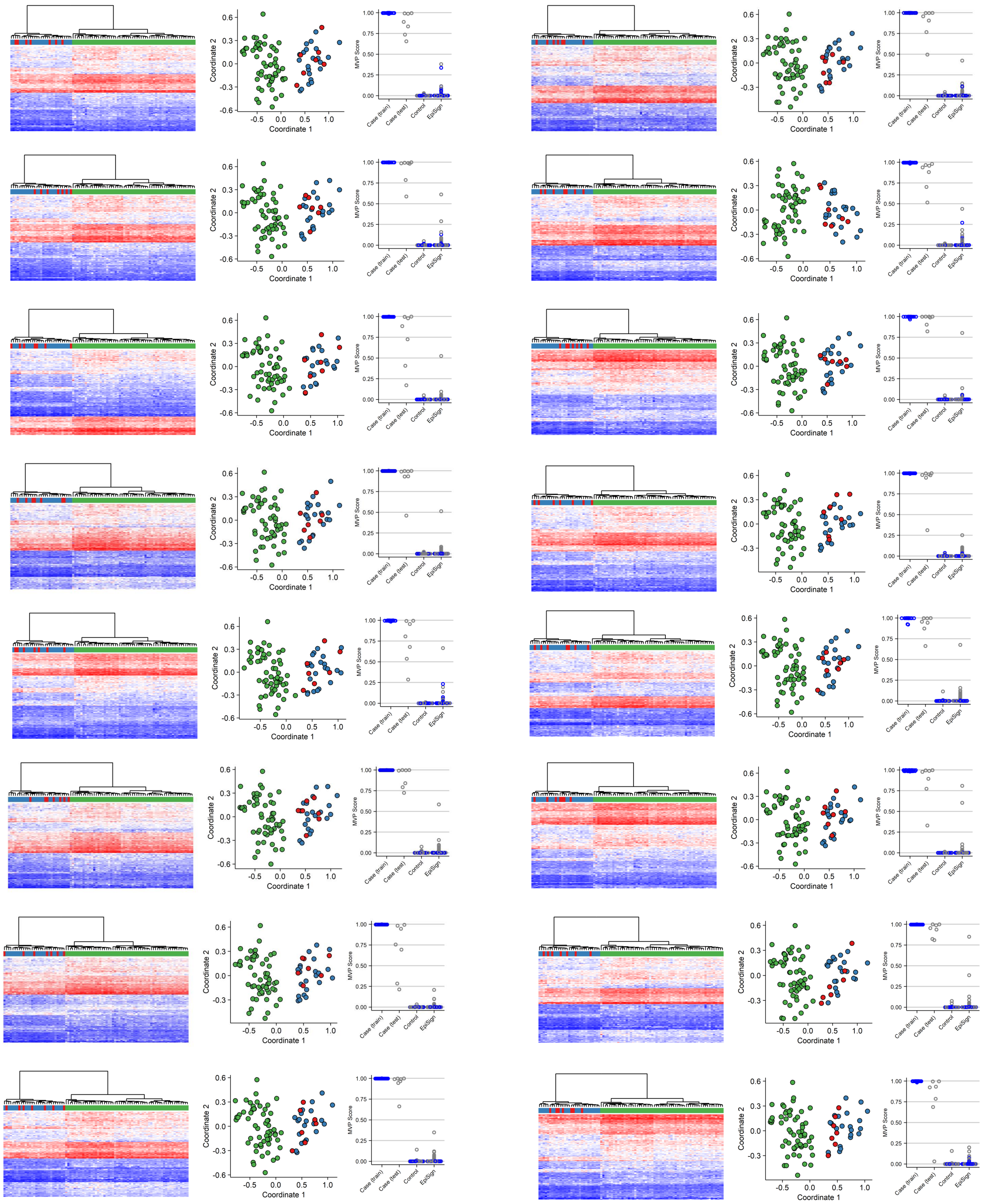

Supplementary Figure 2: Leave-25%-out cross validation (Discovery + validation Cohort)

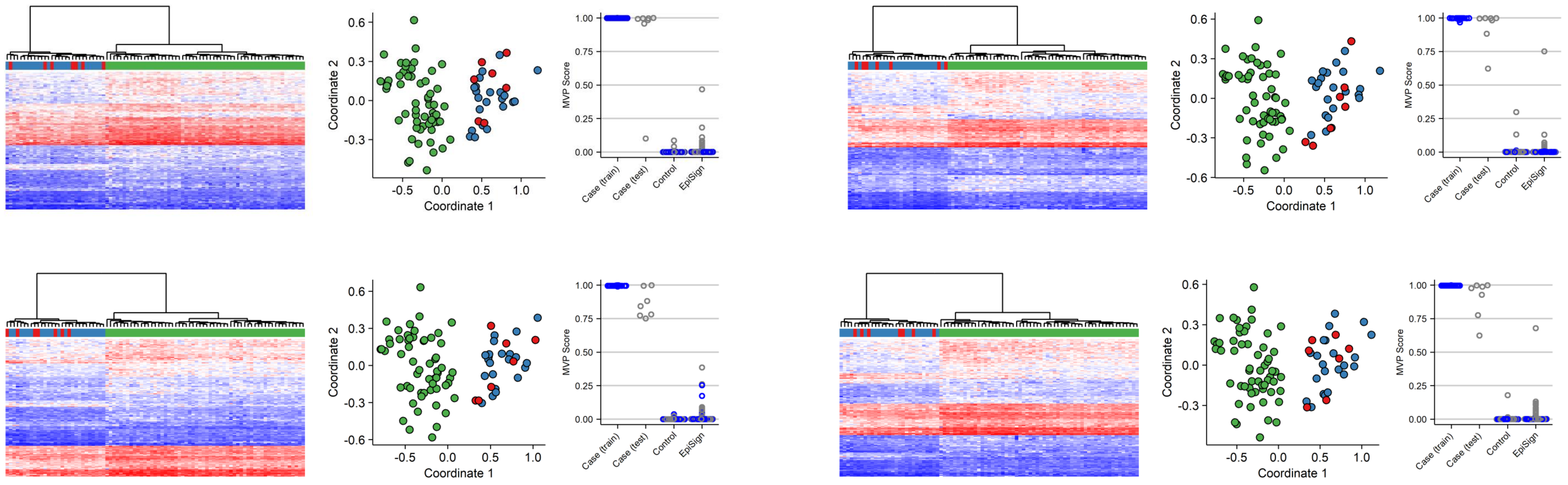

Supplement: Supplementary file 1 [file ijms-23-13664-s001.zip › Supplementary Figures - kopie.pdf]
